# Clinical characteristics and treatment patterns of pregnant women with hypertension in primary care in the Federal Capital Territory of Nigeria: cross-sectional results from the hypertension treatment in Nigeria Program

**DOI:** 10.1186/s12884-023-05723-1

**Published:** 2023-06-03

**Authors:** Zainab Mahmoud, Ikechukwu A. Orji, Gabriel L. Shedul, Kasarachi Aluka-Omitiran, Nanna Ripiye, Blessing Akor, Helen Eze, Tunde Ojo, Guhan Iyer, Abigail S. Baldridge, Lisa R. Hirschhorn, Mark D. Huffman, Dike B. Ojji

**Affiliations:** 1 Washington University, 660 S Euclid Ave, Campus Box 6068, St Louis, MO 63110 USA; 2 Cardiovascular Research Unit, University of Abuja and University of Abuja Teaching Hospital, Gwagwalada, Abuja, Nigeria; 3 Northwestern University Feinberg School of Medicine, Chicago, IL USA; 4 Robert J Havey Institute for Global Health, Chicago, IL USA; 5 The George Institute for Global Health, Sydney, Australia; 6 Faculty of Clinical Sciences, University of Abuja, Abuja, Nigeria

**Keywords:** Hypertension, Pregnancy, Preeclampsia, Maternal mortality

## Abstract

**Background:**

Hypertensive disorders of pregnancy, including hypertension, are a leading cause of maternal mortality in Nigeria. However, there is a paucity of data on pregnant women with hypertension who receive care in primary health care facilities. This study presents the results from a cross-sectional analysis of pregnant women enrolled in the Hypertension Treatment in Nigeria Program which is aimed at integrating and strengthening hypertension care in primary health care centres.

**Methods:**

A descriptive analysis of the baseline results from the Hypertension Treatment in Nigeria Program was performed. Baseline blood pressures, treatment and control rates of pregnant women were analysed and compared to other adult women of reproductive age. A complete case analysis was performed, and a two-sided p value < 0.05 was considered statistically significant.

**Results:**

Between January 2020 to October 2022, 5972 women of reproductive age were enrolled in the 60 primary healthcare centres participating in the Hypertension Treatment in Nigeria Program and 112 (2%) were pregnant. Overall mean age (SD) was 39.6 years (6.3). Co-morbidities were rare in both groups, and blood pressures were similar amongst pregnant and non-pregnant women (overall mean (SD) first systolic and diastolic blood pressures were 157.4 (20.6)/100.7 (13.6) mm Hg and overall mean (SD) second systolic and diastolic blood pressures were 151.7 (20.1)/98.4 (13.5) mm Hg). However, compared to non-pregnant women, pregnant women had a higher rate of newly diagnosed hypertension (65.2% versus 54.4% p = 0.02) and lower baseline walk-in treatment (32.1% versus 42.1%, p = 0.03). The control rate was numerically lower among pregnant patients (6.3% versus 10.2%, p = 0.17), but was not statistically significant. Some pregnant patients (8.3%) were on medications contraindicated in pregnancy, and none of the pregnant women were on aspirin for primary prevention of preeclampsia.

**Conclusions:**

These findings indicate significant gaps in care and important areas for future studies to improve the quality of care and outcomes for pregnant women with hypertension in Nigeria, a country with the highest burden of maternal mortality globally.

## Background

Hypertensive disorders of pregnancy (HDP) including preeclampsia and hypertension are the leading cause of maternal deaths in Nigeria, and as a country Nigeria accounts for 29% of global maternal deaths [1, 2]. In a nationwide analysis of 76,563 deliveries across Nigeria, HDP accounted for 32% of maternal deaths and most are preventable with timely and effective care [3, 4]. In addition to the risk of maternal deaths, HDP are also associated with increased long-term risks for cardiovascular disease, including stroke, heart failure, and myocardial infarction as well as increased risk of adverse foetal outcomes such as preterm birth, low birth weight, neonatal death and worse long-term cardiometabolic profiles [5–8].

Prevalence of hypertension in Nigeria is estimated at 31%, with suboptimal awareness (29%), treatment (12%), and control (2.8%) rates [9]. An important but understudied subset of patients with hypertension is pregnant women. Previous studies in Nigeria have recruited women from secondary and tertiary care facilities, but data are limited among pregnant women with hypertension who receive care within primary health care facilities. The objective of this study was to analyse pregnant women with hypertension who were enrolled into the Hypertension Treatment in Nigeria Program, and to identify areas for future quality improvement.

## Methods

The Hypertension Treatment in Nigeria (HTN) Program is a prospective, longitudinal type 2 hybrid implementation research study designed to evaluate a multilevel, evidence-based implementation package in the Federal Capital Territory using an interrupted time series design (NCT04158154) across 60 selected primary healthcare centres (PHCs) in 6 area councils of the Federal Capital Territory of Nigeria. The study is aimed at integrating and strengthening hypertension care in primary health care centres in Nigeria. To be eligible, PHCs needed to have two full-time staff and be operational and accessible for the research team to visit and work. Sites were selected using a multistage probability sampling frame allowing for adequate geographic representation including urban and rural sites, and further sites selected after consultation with the Nigerian Federal Ministry of Health and the Federal Capital Territory’s Primary Healthcare Board. All selected sites agreed to participate in the study. The HTN program was designed to implement a culturally and contextually adapted, large-scale, multilevel evidence-based implementation package based on the WHO HEARTS technical package and Kaiser Permanente Northern California model of hypertension control and includes: (1) patient registration and empanelment (health system level); (2) standard treatment protocol (national policy level); (3) encouragement of fixed-dose com- bination therapy (health system level); (4) team-based care (health worker level); and (5) home blood pressure monitoring and health coaching (patient-level) [10]. The methods and baseline results, during which consecutive patients with a diagnosis of hypertension were registered, have been reported [11]. These baseline results represent only the preintervention/control phase of the study (January 2020 to November 2020), the intervention period (December 2020 to December 2023) is yet to be completed.

Participating sites were instructed to measure the blood pressure of all adults aged 18 years and above using a standard approach. Trained healthcare workers used an automated blood pressure monitor (Omron M3; HEM-7131- E, Kyoto, Japan) to take two measurements after a 5-minute rest period with the patient sitting and supported. The study team provided regular monitoring and training to ensure accurate measurements were taken. All consecutive adults with a diagnosis of hypertension, defined as: a history of hypertension, persistently elevated SBP of 140 mmHg or above, persistently elevated DBP of 90 mmHg or above, or use of blood pressure-lowering medications, were registered at their respective primary health care centres. Participants were excluded if they were < 18 years or were prisoners or other detained individuals. Pregnant women were eligible for the program, but based on Nigeria’s national hypertension protocol, women who reported being pregnant at enrolment or during the course of the study were diagnosed and referred to a higher level of care for further treatment.

A descriptive analysis of the baseline results among pregnant women with hypertension was performed to evaluate patients’ characteristics and treatment patterns. Baseline blood pressures were reported as means (standard deviation). Baseline treatment rates (walk-in and walk-out) were calculated by dividing the number of patients taking any blood pressure lowering medication at the time of first visit (at entry and exit) by the total number of patients with hypertension. Control rates defined as systolic and diastolic blood pressure less than 140/90 mmHg were also calculated and reported. Findings were compared to other adult women of reproductive age (18–49 years) using t-tests and Chi-squared statistical tests. A complete case analysis was performed, and a two-sided p value < 0.05 was considered statistically significant.

## Results

From January 2020 to October 2022, 5972 women of reproductive age from 60 primary healthcare centres across six area councils in the Federal Capital Territory of Nigeria, were included in the analysis. 112 (2.0%) participants were identified as being pregnant during enrolment or in the course of the study. Patient demographics, characteristics, and treatment patterns are summarized in Table 1.

Mean (SD) age of pregnant patients was lower than all women of reproductive age (34.3 (6.7) versus 39.6 (6.3) years, p < 0.001). One out of every 5 (20.2%) patients had no formal education. Co-morbidities were rare and similar between groups. Overall mean (SD) first systolic and diastolic blood pressures were 157.4 (20.6)/100.7 (13.6) mm Hg and mean (SD) second systolic and diastolic blood pressures were 151.7 (20.1)/98.4 (13.5) mm Hg. Both were similar between groups. Pregnant patients had a higher rate of newly diagnosed hypertension (65.2% versus 54.4% p = 0.02).

Baseline walk-in treatment was lower among pregnant patients (32.1% versus 42.1%, p = 0.03) as shown in Table 2. The control rate was numerically lower among pregnant patients (6.3% versus 10.2%, p = 0.17), but was not statistically significant. Most commonly used agents in pregnant patients were calcium channel blockers (50.0%) and methyldopa (41.7%), and none were on beta blockers. Methyldopa use was higher in pregnant patients (41.7% versus 4.2%, p = < 0.001). None of the pregnant patients were on aspirin.

## Discussion

This report shows that 2% of reproductive-age women enrolled in the PHC-based hypertension treatment program in Nigeria were pregnant. Even though their blood pressures were high, treatment rates were low, and selection of blood pressure lowering drugs was suboptimal. Low baseline walk-in treatment (32%) and control rates (6.3%) demonstrate a potential to improve treatment and control rates as well as diagnosis among this population.

These findings show how primary care is a central avenue to identify pregnant women at high risk of maternal morbidity who can then be referred on to either secondary or tertiary care facilities for further care in accordance with the national treatment protocol [12]. Hypertension including chronic hypertension, as well as gestational hypertension, white-coat hypertension and transient hypertension have all been identified as risk factors for preeclampsia [13–17]. In a large systematic review that included 25,356,688 pregnancies in 90 studies across in 27 countries, the pooled rate of preeclampsia amongst patients with chronic hypertension was 16% (95% confidence interval: 12.6 − 19.7%) compared to 3.1% (95% confidence interval: 3.1% − 3.1%) in those without hypertension [18]. This underscores the need to identify, counsel, treat and monitor all pregnant women with elevated blood pressure during pregnancy.

Treatment of chronic hypertension during pregnancy has been demonstrated to improve both maternal and foetal outcomes [19]. However, in spite of the high burden of hypertension, worse outcomes and proven maternal and foetal benefits of hypertensive treatment in pregnancy, pregnant women are largely excluded from PHC-focused hypertension treatment programs. In a hypertension screening study in Kenya, 4% of women screened were pregnant [20]. In large-scale hypertension treatment programs in South America and China, pregnant women are excluded from the programs [21, 22]. While pregnant women were not excluded from our HTN study, in keeping with the Nigeria’s national hypertension protocol, pregnant women who were diagnosed at PHCs with hypertension were referred to secondary or tertiary centres for further care. Although this may present a barrier to care for pregnant women, complexities of managing pregnant women with hypertension including the need for foetal monitoring, require expertise that is beyond the capacity of the staff at a primary health care centre.

Some pregnant patients (8.3%) were on ACE inhibitors, angiotensin receptor blockers (ARB) or diuretics. These medications are absolutely (ACE/ARBs) or relatively (thiazide diuretics) contraindicated during pregnancy and highlight potential area for training needs amongst patients and healthcare workers to ensure adequate counselling and contraception for women of reproductive age taking these drugs. In pregnant patients, widely established first-line treatment is monotherapy with labetalol, nifedipine or methyldopa [4]. In nonpregnant reproductive-age women on ACE/ARB, appropriate counselling on maternal, foetal risk and the potential teratogenic effects as well as the use of reliable contraception is recommended [23].

Notably, none of the pregnant women were on aspirin for prevention of preeclampsia, even though some might have qualified for this according to the recommendations of the World Health Organization, which identifies women with hypertension as high risk for preeclampsia and recommends prophylactic use of aspirin after 12 weeks’ gestation [24]. Aspirin has been proven to be highly effective and thus universally recommended for the prevention of preeclampsia in patients with hypertension. [24–26].

These findings indicate significant gaps in care and important areas for future studies to improve care and outcomes for pregnant women in Nigeria. It also highlights the potential drawbacks of excluding pregnant women from large scale hypertension treatment programs.

## Limitations

This study has important limitations that should be noted. While the study had geographic representation including urban and rural sites, it was limited to only the capital state in Nigeria and results may not necessarily be generalizable to other states in Nigeria. Secondly, the treatment and control rates presented here represent the baseline visits and may change over time. Third, the pregnancy status of patients was determined through questioning or volunteered by patients. Confirmation of Pregnancy by testing and assessment of gestational age were not performed, therefore gestational age of the pregnancy cannot be accurately ascertained. Fourth, although there were 5972 women of reproductive age within this study, the results and conclusions on characteristics and treatment patterns in the pregnant cohort are limited by sample size and inadequate longitudinal follow up.

## Conclusion

This study examined characteristics and treatment patterns of pregnant women in PHCs within the HTN program in Nigeria. Findings show low treatment and control rates, and suboptimal selection of blood pressure lowering drugs. The indicate significant gaps in care and important areas for future studies to improve the quality of care and outcomes for women contemplating pregnancy and pregnant women with hypertension in Nigeria, a country with the highest burden of maternal mortality globally.

**Table 1:** Baseline Demographics, Clinical Characteristics, Blood Pressures, Overall and By Pregnancy Status

Variables	Overall (*N* = 5972)	Not Pregnant (*N* = 5860)	Pregnant (*N* = 112)	*P* value
Age* (years), mean (SD)	39.6 (6.3)	39.7 (6.2)	34.3 (6.7)	< 0.001
Education [n (%)]				0.57
Never attended school	1206 (20.2%)	1187 (20.3%)	19 (17.0%)	
Grade School	3349 (56.0%)	3281 (56.0%)	68 (60.7%)	
College/University	1417 (23.7%)	1392 (23.8%)	25 (22.3%)	
History of Diabetes [n (%)]	126 (2.1%)	124 (2.1%)	2 (1.8%)	0.83
History of Smoking [n (%)]	18 (0.3%)	18 (0.3%)	0 (0%)	1
History of Chronic Kidney Disease [n (%)]	7 (0.1%)	7 (0.1%)	0 (0%)	1
Prior Heart Attack or Stroke [n (%)]	22 (0.4%)	22 (0.4%)	0 (0%)	1
Timing of Hypertension [n (%)]				0.02
Pre-Existing Diagnosis	2709 (45.4%)	2670 (45.6%)	39 (34.8%)	
New Diagnosis at the time of Registration	3263 (54.6%)	3190 (54.4%)	73 (65.2%)	
First SBP (mmHg), mean (SD)	157.4 (20.6)	157.4 (20.6)	158.3 (20.7)	0.65
Second SBP (mmHg), mean (SD)	151.7 (20.1)	151.7 (20.1)	153.8 (19.4)	0.27
First DBP (mmHg), mean (SD)	100.7 (13.6)	100.7 (13.5)	101.0 (17.1)	0.84
Second DBP (mmHg), mean (SD)	98.4 (13.5)	98.4 (13.5)	100.0 (16.4)	0.30
Controlled (SBP < 140, DBP < 90) [n (%)]	605 (10.1%)	598 (10.2%)	7 (6.3%)	0.17

SD, standard deviation *Age restricted between 18 and 49 years

**Table 2:** Hypertension Treatment Status, Overall and By Pregnancy Status

Variables	Overall (*N* = 5972)	Not Pregnant (*N* = 5860)	Pregnant (*N* = 112)	*P* value
Walk-in Treatment [n (%)]	2503 (41.9%)	2467 (42.1%)	36 (32.1%)	0.03
Drug Class [n (%)]				< 0.001
Calcium Channel Blockers	1871 (74.8%)	1853 (75.1%)	18 (50.0%)	
ACE/ARB/Diuretics	339 (13.5%)	336 (13.6%)	3 (8.3%)	
Beta Blockers	13 (0.5%)	13 (0.5%)	0 (0%)	
Aspirin	20 (0.8%)	20 (0.8%)	0 (0%)	
Methyldopa/Other	260 (10.4%)	245 (4.2%)	15 (41.7%)	
Walk-out Treatment [n (%)]º	5319 (89.1%)	5248 (89.6%)	71 (63.4%)	< 0.001
Drug Class [n (%)]				
Calcium Channel Blockers	4998 (94.0%)	4953 (94.4%)	45 (63.4%)	
ACE/ARB/Diuretics	1295 (24.3%)	1284 (24.5%)	11 (15.5%)	
Beta Blockers	5 (0.09%)	5 (0.1%)	0 (0%)	
Aspirin	6 (0.1%)	6 (0.1%)	0 (0%)	
Methyldopa/Other	114 (2.1%)	94 (1.8%)	20 (28.2%)	
New Treatment	3253 (54.5%)	3208 (54.7%)	45 (40.2%)†	
No Treatment	216 (3.6%)	185 (3.2%)	31 (27.7%)	
Stopped Treatment	315 (5.3%)	305 (5.2%)	10 (8.9%)	
Continued/Altered Treatment	2503 (36.6%)	2162 (36.9%)	26 (23.2%)†	

^o^out of 8 pregnant women, 3 were prescribed ARB and 5 were prescribed diuretics. †4 previously unmedicated pregnant patients were prescribed contraindicated medications; 4 previously medicated patients were prescribed contraindicated medications; walk-in treatment and walk-out treatment rates are the rates at the start and at the end of patients’ baseline visits respectively

## Data Availability

The datasets used and analysed during the current study available from the corresponding author on reasonable request.
